# MetaMembranes for the Sensitivity Enhancement of Wearable Piezoelectric MetaSensors

**DOI:** 10.3390/s22051909

**Published:** 2022-03-01

**Authors:** Saman Farhangdoust, Gary Georgeson, Jeong-Beom Ihn

**Affiliations:** 1 Postdoctoral Research Associate, College of Engineering and Computing, Florida International University, Miami, FL 33174, USA; 2 Boeing Research and Technology, Seattle, WA 98108, USA; gary.e.georgeson@boeing.com (G.G.); jeong-beom.ihn@boeing.com (J.-B.I.)

**Keywords:** MetaMem, metamembrane, piezoelectric pressure sensor, metamaterial, wearable metasensor, auxetic, kirigami

## Abstract

The low stretchability of plain membranes restricts the sensitivity of conventional diaphragm-based pressure and inflatable piezoelectric sensors. Using theoretical and computational tools, we characterized current limitations and explored metamaterial-inspired membranes (MetaMems) to resolve these issues. This paper develops two MetaMem pressure sensors (MPSs) to enrich the sensitivity and stretchability of the conventional sensors. Two auxetic hexagonal and kirigami honeycombs are proposed to create a negative Poisson’s ratio (NPR) in the MetaMems which enables them to expand the piezo-element of sensors in both longitudinal and transverse directions much better, and consequently provides the MPSs’ diaphragm a higher capability for flexural deformation. Polyvinylidene fluoride (PVDF) and polycarbonate (PC) are considered as the preferable materials for the piezo-element and MetaMem, respectively. A finite element analysis was conducted to investigate the stretchability behavior of the MetaMems and study its effect on the PVDF’s polarization and sensor sensitivity. The results obtained from theoretical analysis and numerical simulations demonstrate that the proposed MetaMems enhance the sensitivity of pressure sensors up to 3.8 times more than an equivalent conventional sensor with a plain membrane. This paper introduces a new class of flexible MetaMems to advance wearable piezoelectric metasensor technologies.

## 1. Introduction

**Figure 1:**
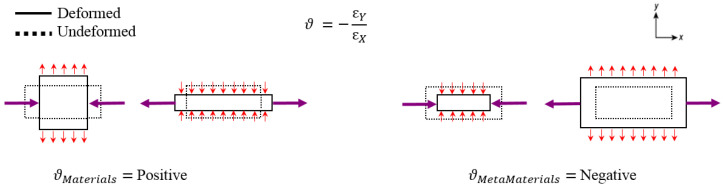
Schematic of deformed and undeformed states of both usual (**left**) and metamaterials (**right**).

Sometimes, conventional piezoelectric sensors have sensitivity limitations, owing to their intrinsic lack of stretchability [1,2,3,4]. In order to enhance stretchability and mechanical compliance, metamaterial-inspired substrates for piezoelectric devices are rapidly growing and becoming widespread [5,6]. Metamaterials are artificial structures which provide unusual mechanical properties with regard to energy absorption, mass, density, deformation, static modulus, smart functionality, and negative Poisson’s ratio (NPR) [6]. During the past decade, there has been a tremendous interest in the use of metamaterial in 1D, 2D, and 3D structures such as: lenses, photonic crystals for light, phononic crystals for sound, and soft acoustic metamaterials [7,8,9]. Poisson’s ratio defines the ratio between two characteristics of the transverse and longitudinal strain of a structure, and NPR behavior has been discovered in auxetic materials that expand (contract) in the transverse direction when stretched (compressed), instead of usual materials (Figure 1) [10,11,12,13,14]. Such an auxetic behavior is found in some hexagonal [15,16,17,18,19,20] and kirigami honeycombs [21,22,23,24,25,26,27]. Auxetic-inspired designs for flexible membranes and substrates are attracting growing attention in developing the next generation of highly efficient piezoelectric sensors and harvesters.

Uniaxial kirigami patterns have been experimentally studied by Hu et al. to improve piezoelectric material stretchability and compliance [28]. A three-dimensional hexagonal honeycomb was studied by Khan and Khan [29] for hydrophone piezoelectric applications. Fey et al. [30] fabricated a two-dimensional auxetic hexagonal lattice from a PZT piezo-ceramic, exhibiting a strain amplification by a factor of 30–70 compared to PZT bulk material. Farhangdoust [31] performed a finite element analysis to investigate the power enhancement of a piezoelectric cantilever energy harvester in which the cantilever beam uses a re-entrant hexagonal auxetic structure. His simulation result showed that the auxetic cantilever beam excited by a harmonic acceleration at a low frequency was able to produce an electric power 2.5 times that of the power produced by the equivalent plain cantilever beam energy harvester. Khan et al. [32] investigated the elastic, dielectric, and piezoelectric properties of hexagonal honeycomb for light-weight piezoelectric sensors and actuators. Very recently, metamaterial-based substrate (MetaSub) was introduced by Farhangdoust et al. [4] for the power enhancement of piezoelectric energy harvesters in which the MetaSub design was made by a combination of uniaxial kirigami and hexagonal patterns to increase the planar stretchability of the substrate. In a myriad of biomedical and wearable health monitoring applications, metamaterial-inspired membranes for implantable strain sensors have gained great attention, as they display great potential for continuous health monitoring. A biaxial pattern of kirigami honeycomb has been used as a substrate for a biomorph piezoelectric harvester by Li et al. [33]. This could enhance the power output by 2.76 times in comparison with an equivalent plain substrate. A hexagonal honeycomb pattern was also developed by Farhangdoust et al. [10] for a stretchable sensor used for the continuous monitoring of structures. Sun et al. [34] used a uniaxial kirigami honeycomb to increase piezoelectricity. It was experimentally found that the kirigami-based sensor improved the voltage output 2.6 times more than a conventional strain sensor. It has been reported that a uniaxial kirigami graphene electrode exhibits a controllable stretchability and strain-insensitive electrical performance up to 240% stretching [35,36]. Sun et al. [37] increased the flexibility performance of wearable sensors by using a uniaxial kirigami honeycomb. A motion artifact-free sensing platform using a kirigami-patterned mesh structure has been carried out by Lee et al. [38] to make a multi-axially stretchable sensor.

Although most of the substantial features of metamaterials have recently been studied for the strain-type of piezoelectric sensors and harvesters, the application of metamaterial-inspired membranes (MetaMem) for inflatable and pressure sensors is still in the early research stage [39]. In a low-pressure regime, the low sensitivity output is an important challenge of conventional pressure sensors (CPSs) using capacitive and piezoresistive measurement principles [39]. In this research, to address this challenge, a MetaMem pressure sensor (MPS) was developed as a highly sensitive alternative to the CPS. Two honeycombs of auxetic hexagonal and biaxial kirigami were exploited to develop a next generation of highly stretchable MetaMems for the sensitivity enhancement of diaphragm-based pressure sensors. To achieve this goal, two MetaMems, as well as a plain membrane, were employed to analyze three pressure sensors by both theoretical and simulation techniques. We first used a CPS comprised of a PVDF layer bonded to a plain membrane to simulate a computational model to characterize the useable frequency range as a function of the natural frequency of the pressure sensor. Accordingly, the two MetaMems were utilized to demonstrate the sensitivity enhancement of the MPSs which depended on the desired stretchability caused by the negative Poisson’s ratio behavior of the proposed MetaMems.

## 2. Design

**Figure 2:**
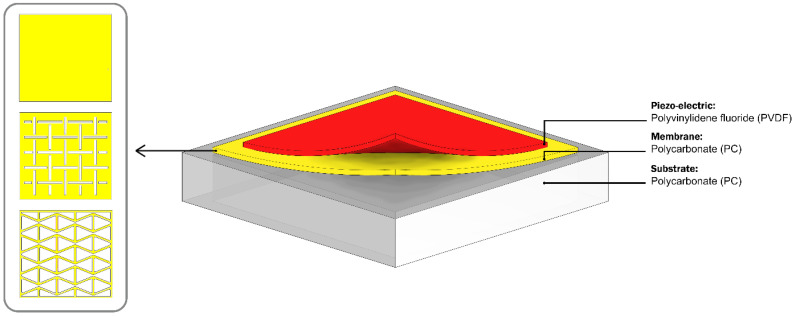

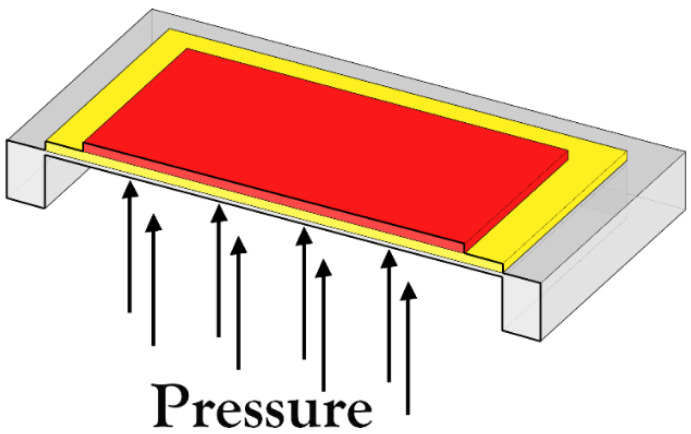
Scheme of the CPS model.

Piezoelectric materials transform mechanical energy into electrical signals when used as a sensor. This is called the direct piezoelectric effect and is expressed using the constitutive equations of Equation (1) [6]:

(1) $$S_{ij}=k_{ijkl}^{E}\sigma_{kl}+d_{kij}E_{k}\;D_{i}=d_{ikl}\sigma_{kl}+\varepsilon_{ik}^{T}E_{k}$$

where *D*, $k^{E}$, σ, and $\varepsilon^{T}$ denote the electrical charge density, compliance under a constant electrical field, applied stress vector, and the dielectric permittivity, respectively. *S*, *d*, *E* and $d^{t}$, also represent the strain, direct piezoelectric effect matrix, electric field, and converse piezoelectric effect, respectively. Figure 2 shows the design for three piezoelectric sensors. The CPS consists of three main components: substrate, membrane, and piezo-element. The substrate has a hole punched from the backside, and is attached to the membrane and piezo-element from the front side, respectively. As the cross section of the CPS is shown in Figure 2, the thin layer of substrate bonded to the membrane acts as a diaphragm film to prevent any direct pressure penetration to the piezo-element from the backside of the sensor. As a result, the strain response of the membrane, and accordingly the stress response of the piezo-element, were analyzed by applying a harmonic pressure to the backside of the substrates. As stated earlier, polyvinylidene fluoride (PVDF) was selected as a preferable piezo-element to use due to its natural flexibility and compatibility. Polycarbonate (PC) was also selected as the material for the substrate and membrane.

**Figure 3:**
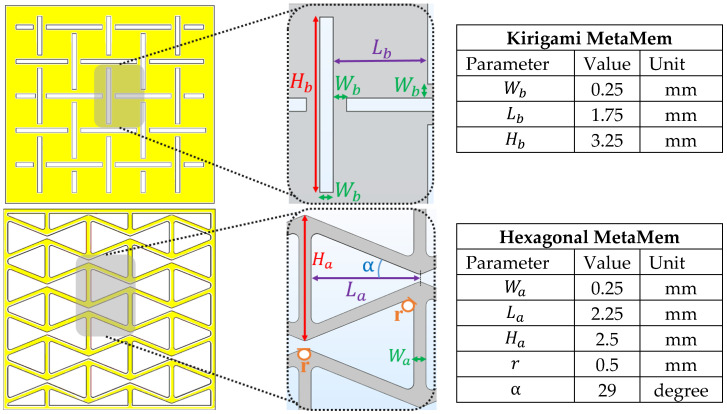
Design of the two MetaMem models; kirigami (**top**), hexagonal (**bottom**).

As shown in Figure 2, to evaluate the stretchability investigation of MetaMems, two distinctive auxetic hexagonal and biaxial kirigami honeycombs were also modeled as the same size as the plain membrane used in the CPS. The dimension and geometric parameters of the CPS model are listed in Table 1. Figure 3 shows the details of the two proposed auxetic hexagonal and biaxial kirigami honeycombs.

For the computational analysis, the material properties of different components of the pressure sensors are listed in Table 2.

**Figure 4:**
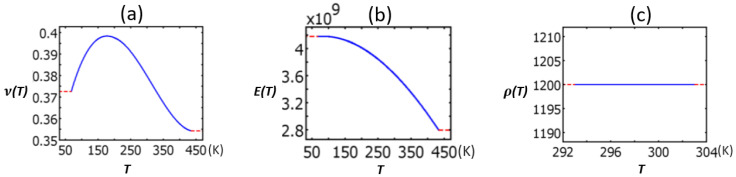
Polycarbonate’s Poisson’s ratio (**a**), elastic modulus (**b**), and density (**c**).

In Table 2, polycarbonate’s Poisson’s ratio (a), elastic modulus (b), and density (c) as functions of temperature were taken from the material library of the COMSOL Multiphysics 3.5a software (Figure 4) [40].

## 3. Finite Element Analysis

**Figure 5:**
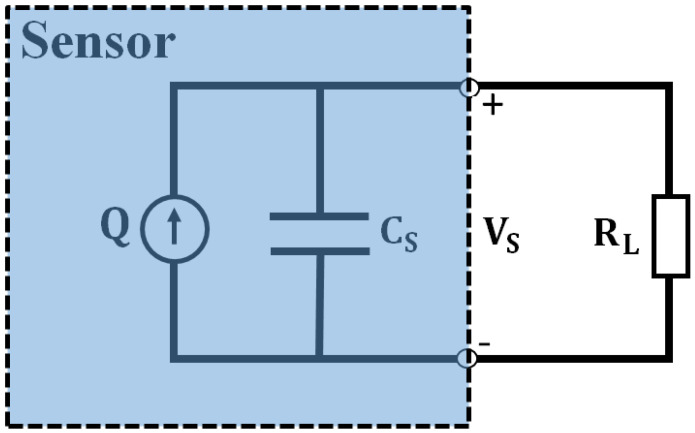
Equivalent circuit model for a piezoelectric sensor.

The finite element analysis (FEA) was employed to investigate the sensitivity enhancement of the two proposed MPSs. To this end, three designs of pressure sensors proposed in the previous section (Figure 2 and Figure 3) were simulated in the three-dimensional geometrics module of the COMSOL Multiphysics 3.5a software. Table 2 was used for material and mechanical properties. In the electrical circuit module of the software, the piezoelectric sensors were modeled as a charge source (Q) in parallel with a capacitor (C_S_) (Figure 5). In addition, the piezoelectric terminals were determined in the electrostatics module of the software. These modules were coupled together to develop and evaluate the three proposed sensors using the frequency domain study of the COMSOL Multiphysics 3.5a software. Accordingly, with regard to the stretchability capacity of the membrane and MetaMems, the voltage output of the three pressure sensors was studied for different sensors and fabrication parameters including resonance frequency, load resistance of the piezo-element, amplitude and frequency of the applied pressure, and the thickness of the membrane/MetaMems.

**Figure 6:**
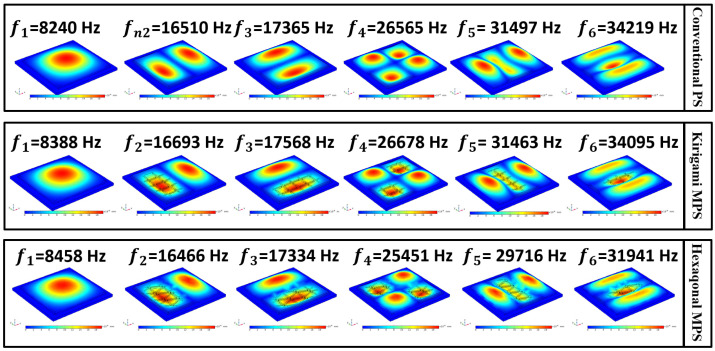
First natural frequencies and mode shapes of the three sensors.

The first five natural frequencies and mode shapes of three sensors were carried out using the eigenfrequency study of the COMSOL Multiphysics 3.5a software. Figure 6 shows the FEM results for three models of CPS with a plain membrane, MPS with a kirigami MetaMem, and hexagonal MPS with a hexagonal MetaMem. As shown in this Figure, the first bending natural frequency of CPS, kirigami MPS, and hexagonal MPS take place at 8240 (Hz), 8388 (Hz), and 8458 (Hz).

**Figure 7:**
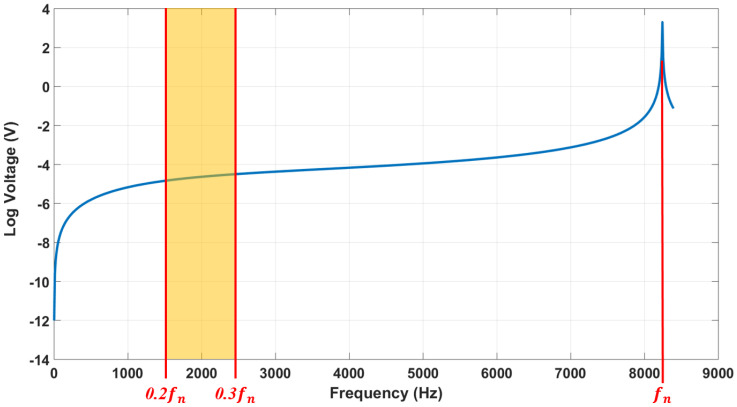
The Log voltage against frequency for the CPS.

As a general rule in pressure sensors, the frequency response of a recessed diaphragm system will be useable from 20% to 30% of the resonance frequency f_n_ [41]. There is a point at 20% of the resonance frequency f_n_ where the sensor’s sensitivity rises about 0.5 dB (5%). Similarly, the sensor’s sensitivity increases about 1dB (10%) at 30% of the resonance frequency f_n_. Hence, as an indicator for frequency analysis in computational study, the preferable frequency range of the models can be defined between those two points of 0.2f_n_ and 0.3f_n_ which are, respectively, 1648 (Hz) and 2472 (Hz). Figure 7 illustrates the log voltage against frequency for the CPS.

**Figure 8:**
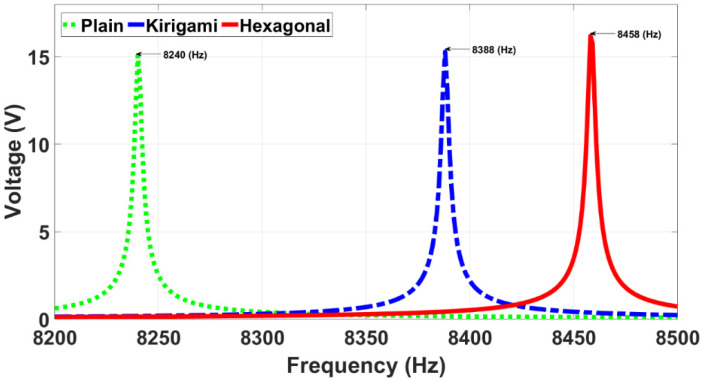
Simulated voltage output against frequency. All points use the same excitation of 5 Pa.

As a preliminary electrical characterization, the voltage output generated by the three simulated sensor models is shown in Figure 8 for when the sensors were subjected to a harmonic pressure. In this Figure, it is clearly identifiable that the resonance frequency is dependent upon membrane design, and ranges from 8240 (Hz) to 8458 (Hz). According to those resonance frequencies, the hexagonal and kirigami MPS models generated voltage outputs of 16.2 (V) and 15.3 (V), respectively, showing a remarkable voltage enhancement in comparison with the CPS that generated 15.0 (V).

To investigate the performance of sensor simulation models, a voltage index (VI) was defined to calculate a normalized voltage output of the proposed auxetic hexagonal MPS and kirigami MPS, with respect to the voltage output of the CPS (Equation (2)):

(2) $$\mathrm{VI}:\;\frac{{V\;}_{\mathrm{MPS}}-{\;V\;}_{\mathrm{CPS}}}{{\;V\;}_{\mathrm{CPS}}}\times 100$$

Table 3 shows normalized voltage outputs for the proposed MPS models at their resonance frequency and 10 kPa pressure amplitude of excitation. It was demonstrated that the auxetic hexagonal honeycomb enabled the pressure sensor to generate the highest voltage with a VI of 9.64%.

**Figure 9:**
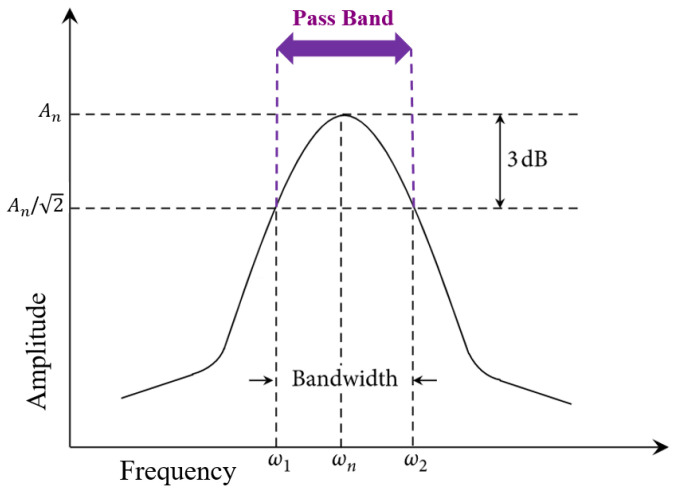
The 3 dB bandwidth indicator.

As a figure of merit (FoM) for resonance behavior, a bandpass filter was applied to the sensors. The bandwidth (BW) of the bandpass filter is usually calculated by $\frac{{\omega \;}_{2}-{\;\omega \;}_{1}}{2{\;\omega \;}_{n}\;}$ to show the maximum data transfer rate of sensors. For 3 dB BW calculations, the signal amplitude of An reduces by 3 dB, i.e., becomes An/$\sqrt{2}$ (Figure 9) [42].

**Figure 10:**
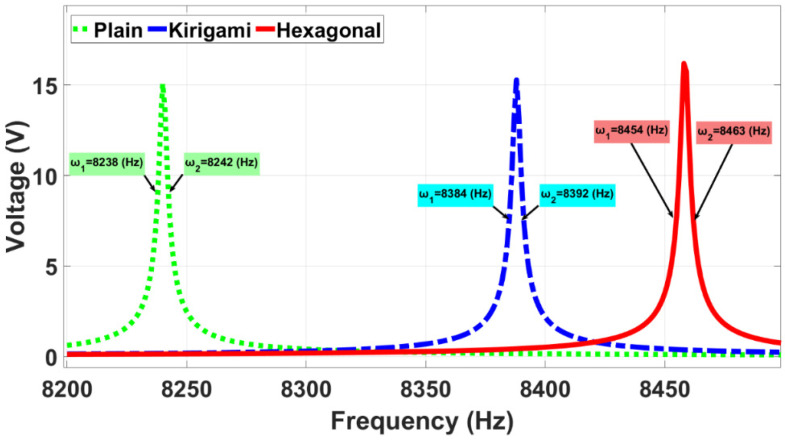
The lower and upper cut-off frequencies of the 3 dB BW for all sensors. All points use the same excitation of 5 Pa.

Figure 10 illustrates the lower cut-off frequency ($\omega_{1}$) as well as the upper cut-off frequency ($\omega_{2}$) of the 3 dB BW for all sensors. The corresponding 3 dB BW of three sensors was tabulated in Table 4. As this table shows, the BW of the proposed auxetic hexagonal MPS and the kirigami MPS were almost two times more than that of the CPS.

**Figure 11:**
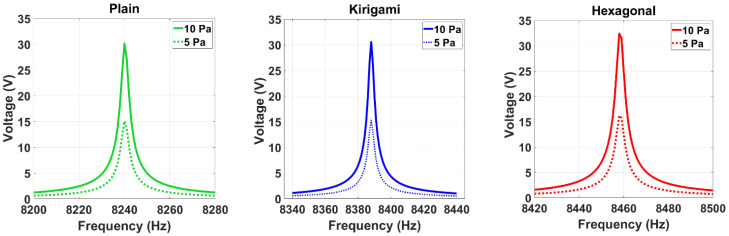
Simulated voltage output against frequency. All points use the same excitation of 5 Pa and 10 Pa.

To investigate the effect of pressure on the sensors’ performances, the voltage output of the three models was determined when the applied pressure doubled. Figure 11 clearly shows that the voltage output increases when the pressure amplitude increases. Furthermore, the auxetic hexagonal MPS provided the best performance at different pressures of excitation.

**Figure 12:**
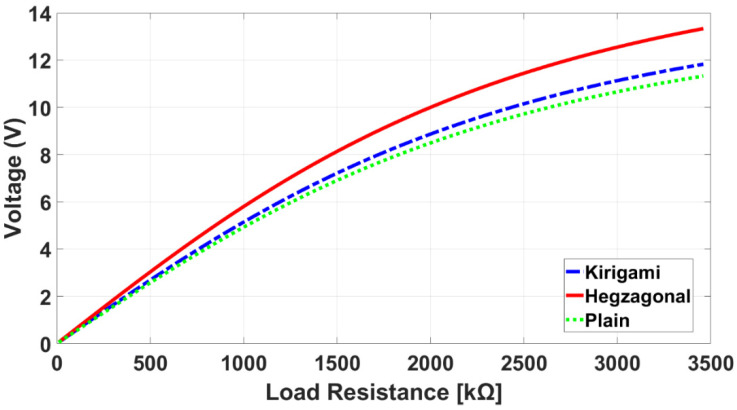
Comparison of simulated voltage output against load resistance for sensors.

As shown in Figure 5, piezoelectric sensors were modeled in parallel with a capacitor and resistor that effectively formed the voltage output. Figure 12 compares the voltage results of three sensors for different load resistances. The voltage first increases with the load resistor and then gradually stabilizes at the voltage output.

Using Kirchoff’s law, the voltage output for the electrical circuit generated across the electrodes of a thin piezo-layer under dynamic bending is [6]:

(3) $$V_{S}=\frac{t_{p}D_{3}}{\varepsilon_{0}\varepsilon_{33}}$$

where $\varepsilon_{0}$ and $t_{p}$ denote the permittivity of free space and the thickness of the PVDF, respectively. Assuming an isotropic and planar behavior for the piezoelectric sensors, the electrical charge density of the sensors can be calculated by the scalar Equation (4):

(4) $$D_{3}=\frac{Q}{A_{p}}=d_{31}\left(\sigma_{11}+\sigma_{22}\right)$$

where A, $\sigma_{11}$, $\sigma_{22}$ and $d_{31}$ denote PVDF area, longitudinal stress, transverse stress, and the piezoelectric constant, respectively. For piezoelectric sensors, the maximum electric power is proportional to the square of the RMS of the voltage output, and takes place at the optimum load resistance ($R_{L}$) (Equation (5)) [6,43].

(5) $$P_{\mathrm{Max}}=\frac{V_{RMS}{}^{2}}{R_{L}}=\frac{{\mathrm{fA}}_{p}t_{p}d_{31}^{2}}{\varepsilon_{0}\varepsilon_{33}}\left(\bar{\sigma_{11}}+\bar{\sigma_{22}}\right)$$

Theoretically, the optimal load resistance ($R_{L}$) matches the internal impedance of the piezo-element and can be calculated by Equation (6) [6].

(6) $$R_{L}=\frac{1}{2{\pi \mathrm{fC}}_{s}}=\frac{t_{p}}{2{\pi f\varepsilon }_{0}\varepsilon_{33}A_{p}}$$

**Figure 13:**
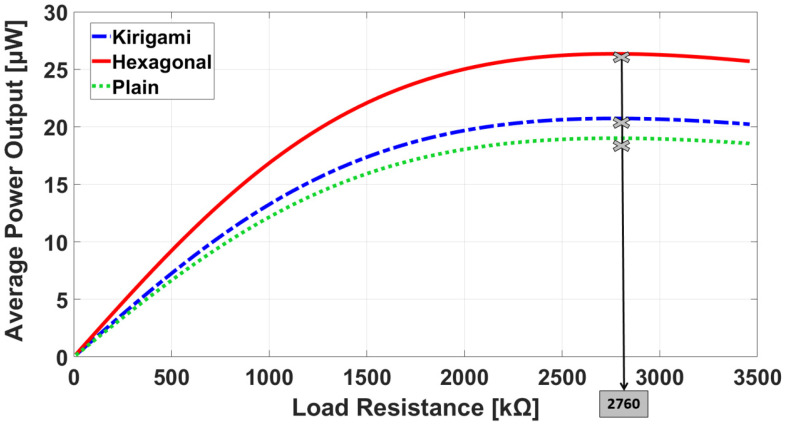
Simulated average power output against load resistance for the sensors.

To find the maximum power output and corresponding load resistance of the sensors, FEA was carried out when a pressure amplitude of 10 kPa was applied to the simulation models. As shown in Figure 13, the maximum power output for the three models took place at the load resistance of 2760 kΩ, which is very close to the theoretical optimum load resistance of 2756 kΩ calculated by Equation (6).

Finite element modelling was used to investigate the benefits of the MetaMems on performance enhancement. It is generally acceptable to use pressure sensors over a frequency range between 0.2f_n_ and 0.3f_n_. As mentioned earlier, 0.2f_n_ and 0.3f_n_ of CPS are 1648 (Hz) and 2472 (Hz), respectively. Furthermore, most micro-electromechanical systems (MEMS) require an appropriate sensitivity in a low-pressure regime, less than 10 kPa [44]. Therefore, the simulation models were developed in pressure range of 0 to 10 kPa at 1648 (Hz).

**Figure 14:**
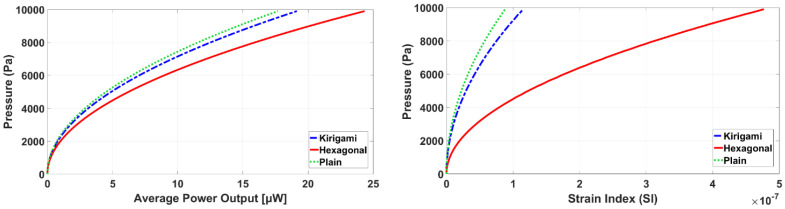
Average power output (**left**) and strain performance (**right**) against pressure all sensors.

The strain performance and power output of sensors for a wide range of pressure amplitudes are presented in Figure 14. Figure 14 left shows the impact of the MetaMems’ mechanism on the power increase is positive, specifically for the hexagonal MPS. In general, the membrane/MetaMem transfers deformation energy of the applied bending over the PVDF, and accordingly polarizes the PVDF to generate electric power output. Since the power output depends on the sum of the axial and lateral stress tensors across the PVDF, a strain index (SI) was studied for all sensors in Figure 14 right. The SI was defined based on a longitudinal and transverse strain of the membrane/MetaMem, ${\left(\bar{\varepsilon_{11}}+\bar{\varepsilon_{22}}\right)}^{2}$. Thus, the better strain performance a sensor has, the more power output its PVDF generates. Figure 14 right clearly shows the hexagonal MetaMem had the best deformation performance in different pressure amplitudes, causing a higher stress concentration across the PVDF in bending pressure, and accordingly greater power output (Figure 14 right).

**Figure 15:**
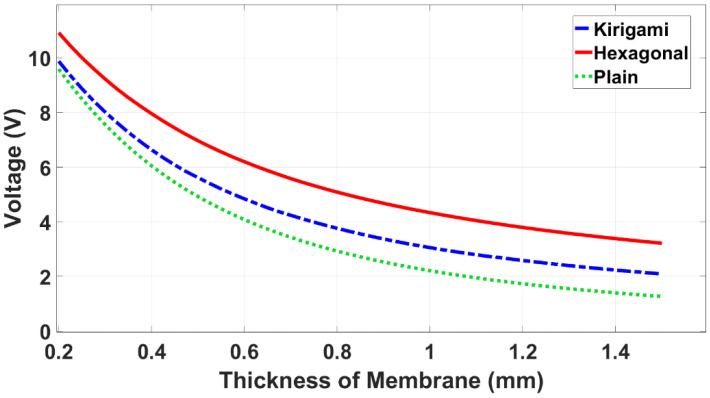
Simulated voltage output against the thickness of the membrane and MetaMems.

As a further design investigation, simulations were performed to study the effect of the thickness of the plain membrane and two MetaMems on the voltage output when the pressure sensors were actuated at 10 kPa, 2760 kΩ, and 1648 (Hz). The results showed that as the thickness of the membrane and MetaMems increased, the voltage output of all three sensor models decreased (Figure 15). This is because the deformation from the substrate was transferred to the piezo-element by the membrane/MetaMem, and therefore, when the thickness of the membrane/MetaMem increased, more strain energy was dissipated in the membrane/MetaMem, with a reduced strain energy transferred to the piezo-element.

## 4. Results and Discussions

The voltage output over strain input is defined as the sensitivity of sensors [45]. To clarify, the membrane/MetaMem transfers strain energy to the piezo-element, and causes polarization across the piezo-element to generate voltage. Equation (7) was used to investigate the effect of the membrane design on the sensor sensitivity, S.

(7) $$S=V/{\left(\bar{\varepsilon_{11}}+\bar{\varepsilon_{22}}\right)}^{2}$$

In this simulation, the sensitivity of the three models were studied at the usable range of pressure amplitudes and frequencies. The optimum values calculated in the previous sections were used as the load resistance and membrane/MetaMem thickness. To predict a magnification factor for the proposed MPSs, their sensitivity was investigated for different excitation frequencies and amplitudes in which the thin diaphragm film of the substrate was subjected to a bending movement caused by a harmonic pressure of 1 to 10 kPa at a frequency range of 1648 (Hz) to 2472 (Hz).

**Figure 16:**
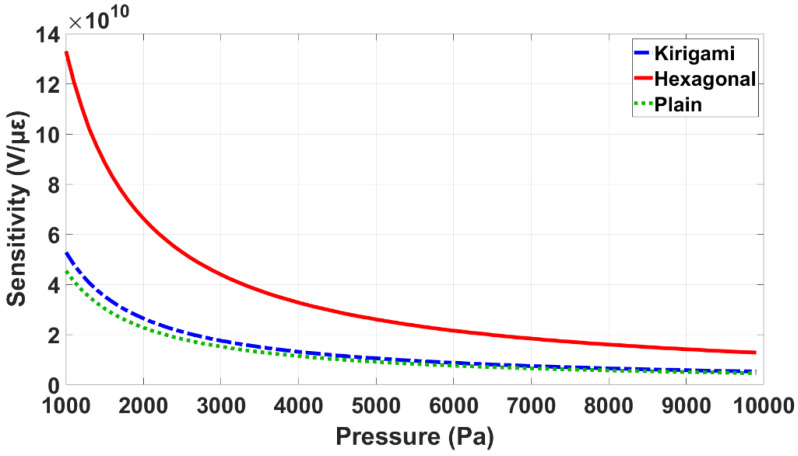
Sensitivity performance of the three simulated models in different pressure amplitudes.

**Figure 17:**
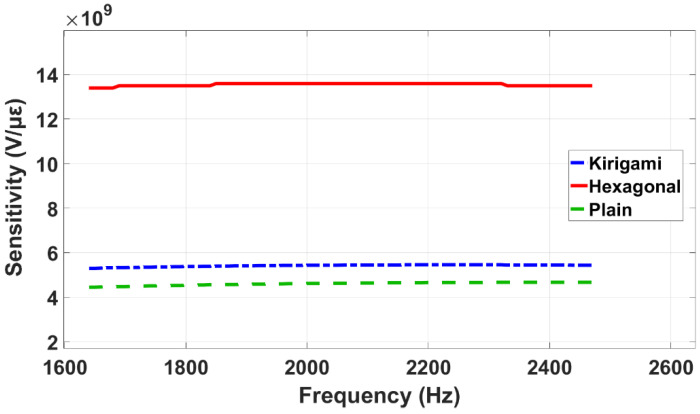
Sensitivity performance against frequency of the three sensor models.

The effects of the pressure amplitude and frequency on the performance of the simulated sensors are illustrated in Figure 16 and Figure 17. As is shown in Figure 16, the sensitivity of the three models decreases with the pressure amplitude. The applied frequency was kept constant at 1648 (Hz) when the pressure amplitude was varied (Figure 16).

Furthermore, as shown in Figure 17, the sensitivity remains constant when the frequency changes between 0.2fn and 0.3fn. When the frequency varied, the pressure amplitude was kept constant at 10 kPa (Figure 17).

**Figure 18:**
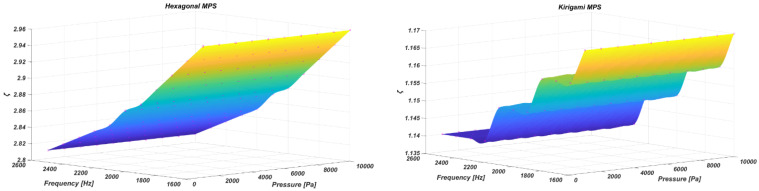
Distribution of the sensitivity gain factor for the hexagonal (**left**) and kirigami (**right**) MPSs.

In Figure 18, the sensitivity of the two proposed MPSs—the auxetic hexagonal and kirigami MetaMems—was compared to an equivalent CPS using a plain membrane. A comparison ratio of ζ is defined as the sensitivity gain factor in order to evaluate the sensitivity performance of the MPSs against the CPS (Equation (8)).

(8) $$\zeta =\frac{S_{MPS}}{S_{CPS}}$$

Figure 18 illustrates the distribution of the sensitivity gain factor for the auxetic hexagonal and kirigami MPSs with varying applied pressure amplitudes and frequencies.

**Figure 19:**
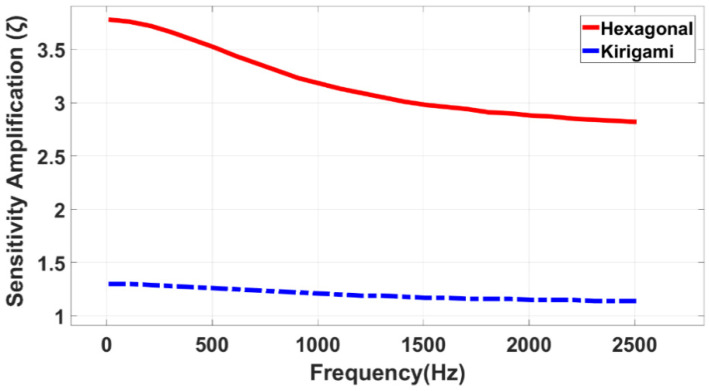
Sensitivity amplification of auxetic hexagonal and kirigami MPSs at different frequencies.

The two contours shown in Figure 18 prove that the sensitivity amplification remains constant across different pressure amplitudes, which means the magnification factor is a function of the membrane/MetaMems geometry of the sensors, and is not dependent on the excitation conditions. As a further evaluation, the sensitivity amplification for both the auxetic hexagonal and kirigami MPSs was investigated at a wide range of frequency. As demonstrated in Figure 19, the sensitivity gain factor for the auxetic hexagonal and kirigami MPSs can reach up to 3.8 and 1.3, respectively.

In order to explore reasons of such remarkable sensitivity enhancement and different reactions to an identical excitation, first the stress distribution across the piezo-elements layer was obtained, and then the strain response of the plain membrane and two metamembranes was investigated and compared. According to our sensitivity definition (Equation (7)), the sensitivity of a pressure sensor depends on the stretchability of its membrane and the voltage generated by its piezo-element. Hence, a simulation was developed using COMSOL Multiphysics 3.5a software to investigate the sensitivity performance of the three sensors when a sinusoidal pressure was applied on the surface of their substrate’s diaphragm (Figure 2) along the Z-axis at 10 kPa and a frequency of 1648 (Hz). The thickness of plain membrane and two MetaMems was also considered to be 0.25 mm.

For the pressure sensors, the voltage output was made by the piezo-element polarization along the Z-axis, and was proportional to the square root of optimal power output (Equation (9)) [3].

(9) $$V_{RMS\;}=\sqrt{R_{L\;}P_{opt}}\;$$

The RMS of the voltage output could be then obtained by substituting Equations (2) and (4) into Equation (10) [4]:

(10) $$V_{RMS\;}=\frac{t_{p}d_{31}}{\varepsilon^{T}}\left(\bar{\sigma_{11}}+\bar{\sigma_{22}}\right)\;$$

**Figure 20:**
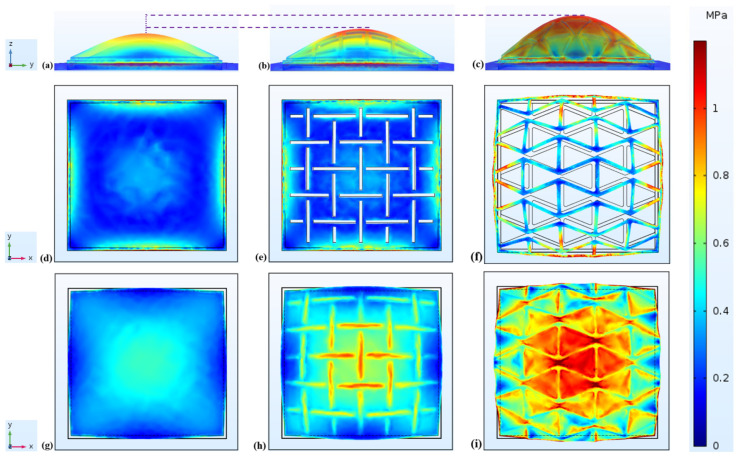
Each column is given to one type of sensor: CPS (**a**,**d**,**g**), kirigami MPS (**b**,**e**,**h**), and hexagonal MPS (**c**,**f**,**i**). The first row shows the displacement and stress distribution of sensors from side view (**a**–**c**). The second row illustrates displacement and stress distribution of membrane/MetaMems from top view (**d**–**f**). The third row displays displacement and stress distribution PVDF from top view (**g**–**i**).

Table 5 summarizes the voltage output for all simulated sensors. The results showed that the MPSs with the MetaMem generated more voltage compared to the CPS with the plain membrane. Since the voltage output is related to the sum of the axial and lateral stress tensors for the sensor’s piezo-element (Equation (8)), the stress distribution across the piezo-element layer was also examined for the sensors in Table 4. This table demonstrates that the mean value of stress across the PVDF of auxetic hexagonal and kirigami MetaMems was greater than the plain CPS. The stress distribution across the PVDF of the three sensors is shown in Figure 20g–i. The black lines were considered to show the original shape, and the deformation in these figures is scaled up by 500 times for a better evaluation. As observed in this figure, most of the area of the PVDF’s surface for the two MPSs experiences a higher level of stress compared to the CPS. The peak stress of 6.64 MPa, 4.98 MPa, and 4.56 MPa were observed in the hexagonal MPS, kirigami MPS, and CPS, respectively. It is worth mentioning that these stress values are sufficiently below the yield strength of both PVDF and PC (30–80 MPa) [46,47,48].

The membrane/MetaMem transfers the stress of the applied bending over the PVDF. Therefore, we need to examine the strain performance of the membrane/MetaMem to understand the stretchability of the MetaMem mechanism and its impact on the PVDF stress distribution and voltage increase.

Furthermore, Figure 20a–c illustrate that the MPSs presents greater displacement along the Z-axis compared to the CPS. The deformations are scaled up by 200 times for clarity. From a structural standpoint, the displacement of the pressure sensors in the Z-axis are caused by the membrane’s expansion in other directions ($\varepsilon_{11}$ and $\varepsilon_{22}$).

Since an auxetic design inherently enables structures to stretch more, we expect that the two auxetic hexagonal and kirigami honeycombs provide a stretchability greater than the plain membrane. The maximum longitudinal and transverse strains for the three simulated models are tabulated in Table 6. As a result, the auxetic hexagonal and kirigami meta-materials demonstrated remarkable longitudinal and transverse strain values. The two proposed honeycombs help the flexible MetaMems to enrich the sensor stretchability and transfer more strain energy to the piezo-element.

In Figure 20d–f it can be also observed that the displacement fields of the metamaterials are severely distorted by the auxetic hexagonal and kirigami honeycombs. In this Figure, the deformation is scaled up by 1000 times for clarity, and the black lines were considered to show the original shape.

There are many techniques to fabricate such pressure sensors. Usually, the fabrication process includes iterations of film depositions, micro-patterning features, and etching to create the desired layers of a sensor. Transfer printing is one of the fabrication methods which enables a combination of materials with different properties onto flexible membranes. Moreover, 3D printing and 4D printing are other techniques that can be achieved through various additive manufacturing processes such as micro-stereolithography, multiphoton lithography, laser chemical vapor deposition (LCVD), laser-induced forward transfer (LIFT), and UV lithography [39,49,50,51,52].

## 5. Conclusions

FEA was performed to determine the sensitivity enhancement of two proposed auxetic hexagonal and kirigami MPSs by using COMSOL Multiphysics 3.5a software. The computational results demonstrated that the 3 dB bandwidth of the MPSs was two times greater than the CPS, and the sensitivity gain factor for the auxetic hexagonal and kirigami MPSs also reached up to 3.8 and 1.3, respectively, when their substrates were subjected to a bending movement. In order to explore reasons of such a remarkable sensitivity enhancement, the stress distribution across the PVDF layer as well as the strain response of the MetaMems were investigated for the two proposed MPSs and, accordingly, their results were compared to the CPS’s output. Since the two auxetic hexagonal and kirigami honeycombs inherently enable MetaMems to stretch better, it was found that the MPSs indicated a higher flexural deformation capability compared to the plain membrane. The elastic energy from the substrate transfers to the PVDF layer by the diaphragm film. Therefore, the stress across the PVDF layer will be increased as much as the strain response of the diaphragm film increases. Numerical results showed that the auxetic hexagonal MetaMem with 0.4 mε enhanced the strain capability of the sensor up to 4 times, compared to plain membrane with 0.1 mε, when they were subjected to a harmonic pressure at a frequency of 1648 (Hz) and 10 kPa. Accordingly, the average stresses of 0.5 MPa and 0.3 MPa were measured across the PVDF of the MPSs and CPS, respectively, enabling the pressure sensor to generate the highest possible voltage output with a normalized voltage index of 9.64%. The finite element modelling also showed that the magnification factor remains approximately constant across different pressure amplitudes, which means it is a function of the MetaMems geometry and does not depend on the excitation conditions. This paper opens up great potential for using MetaMem applications for different flexible sensor systems in wearable technologies.

## Figures and Tables

**Table 1:** Parameter values used in the pressure sensor models.

Part	Parameter	Value	Unit
**Substrate**	$\mathrm{Width},\;W_{s}$	14	mm
	$\mathrm{Length},\;L_{s}$	13.5	mm
	$\mathrm{Thickness},\;t_{s}$	500	μm
	$\mathrm{Diaphragm}’s\;\mathrm{Thickness},\;t_{d}$	125	μm
**Membrane**	$\mathrm{Width},\;W_{m}$	12.125	mm
	$\mathrm{Length},\;L_{m}$	11.5	mm
	$\mathrm{Thickness},\;t_{m}$	250	μm
**Piezo-element**	$\mathrm{Width},\;W_{p}$	11.625	mm
	$\mathrm{Length},\;L_{p}$	11	mm
	$\mathrm{Thickness},\;t_{p}$	250	μm

**Table 2:** Material properties for models.

Material	Property		Value
**Piezo-Element:** **Polyvinylidene fluoride (PVDF)**	$\mathrm{Density},\;\mathrm{kg}/m^{3}$	$\rho_{\mathrm{PVDF}}$	1780
	Load Resistance, k Ω	R	2000
	Compliance Matrix, p/Pa	$s_{11}^{E}$	378
		$s_{33}^{E}$	109
	$\mathrm{Coupling}\;\mathrm{Matrix},\;10^{-12}$ C/N	$d_{31}$	13
		$d_{32}$	14
		$d_{33}$	−33
	Relative Permittivity, --	$\in_{33}$	7.6
**Substrate and Membrane:** **Polycarbonate (PC)**	$\mathrm{Density},\;\mathrm{kg}/m^{3}$	$\rho_{\mathrm{PC}}$	ρ(T)
	Poisson’s Ratio	$\nu_{\mathrm{PC}}$	ν(T)
	Young’s Modulus, GPa	$E_{\mathrm{PC}}$	E(T)

**Table 3:** Normalized voltage output of the two proposed auxetic MPS models: kirigami and hexagonal.

Type	f_n_ (Hz)	VI (%)
Kirigami	8388	1.33
Hexagonal	8458	9.64

**Table 4:** 3 dB bandwidth of the proposed sensors.

Type	Amplitude (v)	ω_n_ (Hz)	ω_1_ (Hz)	ω_2_ (Hz)	BW*_MPS_*/BW*_CPS_*
Plain	15.076	8240	8238	8242	1
Hexagonal	16.184	8458	8454	8463	2.19
Kirigami	15.277	8388	8384	8392	1.96

**Table 5:** Voltage output and longitudinal as well as transverse stresses for all simulated models.

Design	$\bar{\sigma_{11}}$, MPa	$\bar{\sigma_{22}}$, MPa	*V_RMS_*, V
Hexagonal	0.5	0.5	13.74
Kirigami	0.3	0.4	11.33
Plain	0.3	0.3	10.61

**Table 6:** Longitudinal and transverse strains for all simulated models.

Design	$\varepsilon_{11}$, με	$\varepsilon_{22}$, με
Hexagonal	468	475
Kirigami	228	236
Plain	188	194

## Data Availability

Requests for access to the data of this research should be made to Saman Farhangdoust.
